# Phylogenetic Patterns of Codon Evolution in the ACTIN-DEPOLYMERIZING FACTOR/COFILIN (ADF/CFL) Gene Family

**DOI:** 10.1371/journal.pone.0145917

**Published:** 2015-12-30

**Authors:** Eileen M. Roy-Zokan, Kelly A. Dyer, Richard B. Meagher

**Affiliations:** Department of Genetics, University of Georgia, Athens, Georgia, United States of America; College of Agricultural Sciences, UNITED STATES

## Abstract

The actin-depolymerizing factor/cofilin (ADF/CFL) gene family encodes a diverse group of relatively small proteins. Once known strictly as modulators of actin filament dynamics, recent research has demonstrated that these proteins are involved in a variety of cellular processes, from signal transduction to the cytonuclear trafficking of actin. In both plant and animal lineages, expression patterns of paralogs in the ADF/CFL gene family vary among tissue types and developmental stages. In this study we use computational approaches to investigate the evolutionary forces responsible for the diversification of the ADF/CFL gene family. Estimating the rate of non-synonymous to synonymous mutations (dN/dS) across phylogenetic lineages revealed that the majority of ADF/CFL codon positions were under strong purifying selection, with rare episodic events of accelerated protein evolution. In both plants and animals these instances of accelerated evolution were ADF/CFL subclass specific, and all of the sites under selection were located in regions of the protein that could serve in new functional roles. We suggest these sites may have been important in the functional diversification of ADF/CFL proteins.

## Introduction

The actin cytoskeleton is one of the most dynamic features in a eukaryotic cell. It is involved in a number of important and diverse cellular processes such as organelle movement, exo- and endocytosis, nuclear trafficking, and chromatin remodeling. A variety of classes of actin binding proteins are found in plants and animals that facilitate the dynamic nature of actin, one of which is encoded by the actin-depolymerizing factor/cofilin (ADF/CFL) gene family. ADF/CFLs comprise a group of relatively small proteins approximately 13–19 kDa in size [1] with a shared conserved structural motif known as the actin-depolymerizing factor homology (ADF-H) domain [2, 3] that is characterized by the presence of five β-strands that lie internal to four or more α-helices [3]. ADF/CFLs were first described as proteins that bind and sever actin monomers from F-actin filaments, but this ADP-actin filament severing activity is only present at low cellular concentrations of ADF/CFL [1, 4, 5]. In contrast, at intermediate concentrations ADF/CFLs have been shown to stabilize filaments, and at high concentrations they promote the nucleation of filaments [5]. As more research is conducted on the ADF/CFL gene family, it is becoming evident that their role within the eukaryotic cell extends well beyond their F-actin filament turnover activities; for instance, ADF/CFL proteins have been implicated in cellular processes that range from membrane and lipid metabolism to mitochondrial dependent apoptosis [6, 7, 4]. The multifaceted and important roles of the ADF/CFL gene family members within a eukaryotic cell resulted in Bernstein and Bamburg [4] to characterize these proteins as “*a functional node in cellular biology*.”

While invertebrate lineages and unicellular eukaryotic organisms typically possess only a single ADF/CFL variant [1], the ADF/CFL gene family underwent an expansion in vertebrate lineages, and consist of three classes of protein variants: CFL1, CFL2, and ADF (also referred to as Destrin). Although most vertebrates have two or more variants of ADF/CFL, only mammals possess an ADF/CFL variant from each of the three classes [8, 9]. All three classes of vertebrate ADF/CFL proteins are spatially and temporally regulated [1]. Within mammals this regulation may even be seen within a single cell as the two non-muscle variants (CFL1 and ADF) are often expressed in different locations in a cell and at different developmental stages [10]. Extensive partitioning of expression is also seen within ADF protein variants across plant lineages. Plant ADF protein variants group into four ancient subclasses (ADF I-IV) [9, 11, 12] that have been conserved in angiosperms for approximately 250 million years [12]. While the number of ADF variants varies among plant species, all angiosperms possess at least one protein variant from each of the four subclasses. Studies in Arabidopsis show that each subclass has its own unique expression profile and there are distinct profiles within some subclasses that predate the divergence of monocots and dicots [12].

The tissue- and temporal-specific partitioning of expression patterns suggests that ADF/CFL protein variants have subfunctionalized, but also may have gained novel functions during their evolutionary history. Mammalian CFL and ADF/Destrin have biochemical differences that are highly suggestive of functional divergence [4]. ADF/Destrin has been shown to have greater monomer sequestering and actin depolymerization activities than CFL1, while CFL1 is a more efficient filament nucleator than ADF/Destrin [13, 14, 4]. Knocking out CFL results in embryonic lethality in mice, whereas knocking out ADF/Destrin results only in minor developmental defects [4]. Additionally, mammalian muscle (CFL2) and non-muscle (CFL1) cofilins interact differently with F-actin, suggesting that these two protein variants may have differing roles in actin filament dynamics [15]. The evidence for functional diversification is also strong for the plant ADF gene family. Even the closely related subclass III Arabidopsis ADF paralogs, ADF5 and ADF9, have contrasting and unique phenotypes that cannot be suppressed through the exogenous expression of its respective paralog [16]. There have also been a few studies that have highlighted the varying and specialized roles of different Arabidopsis subclass I protein variants in pathogen defense. For instance, Arabidopsis plants that are defective in ADF4 are more susceptible to *Pseudomonas* infection than wild-type plants [17]. Interestingly, this role in *Pseudomonas* immunity is unique to ADF4 and the other paralogs do not contribute to this phenotype. Similarly, only ADF2 plays a crucial role in the establishment of nematode infection in Arabidopsis [18].

The goal of this study is to identify the regions of the proteins and the particular amino acid residues that may have facilitated functional diversification and subsequent versatility of the ADF/CFL gene family. To do this, we estimated the rate of selection across the gene family phylogeny and across individual protein sequences. Estimates of selective pressure are often useful when investigating genes that have undergone duplication, as duplicates may experience accelerated rates of evolution due to both relaxed purifying and/or positive selection (reviewed in [19, 20]). Estimating selection across a gene phylogeny has been used to decipher the molecular evolution of important gene families such as the triplicate alpha-globin genes in rodents and MADS-box transcription factors in plants [21, 22]. Here we describe molecular evolutionary analyses of the animal ADF/CFL and plant ADF gene families in order to examine lineage specific conservation or divergence of residues. Our results help identify those protein variants that may have neofunctionalized after duplication and also identify particular amino acids within these divergent proteins that were most likely involved in adaptive evolution. These sites provide a good target for future studies investigating the functional differences between ADF/CFL protein variants.

## Materials and Methods

### Sequence Acquisition and Alignment

We chose to use species that had a fully annotated genome and that represented significant temporal spacing from common ancestry. The following plant species were used: *Physcomitrella patens*, *Selaginella moellendorffii*, *Zea mays*, *Oryza sativa japonica*, *Vitis vinifera*, *Mimulus guttatus*, *Populus trichocarpa*, and *Arabidopsis thaliana* (see S1 Table for divergence times). This includes two model monocot species as well as four dicot species to sample the evolutionary history of angiosperms. Gymnosperms were not included because there were no gymnosperm species that had their complete ADF gene family annotated at the time of our analysis. The non-vascular bryophyte *Physcomitrella patens* and the lycophyte *Selaginella moellendorffii* were used to root the tree. To verify the expression of *S*. *moellendorffii* protein variants RNA was extracted from frond tissue and ADF RNA levels were quantified using qRT-PCR.

For the metazoan ADF phylogeny, we used seven vertebrate species (*Salmo salar*, *Danio rerio*, *Xenopus tropicalis*, *Gallus gallus*, *Sus scrofa*, *Mus musculus*, and *Homo sapiens*) and four invertebrate species (*Ciona intestinalis*, *Branchiostoma floridae*, *Caenorhabditis elegans*, and *Drosophila melanogaster*) (see S2 Table for divergence times). The ADF/CFL from the protist species *Monosiga brevicolis* was included as an outgroup because this choanoflagellate is considered the closest characterized relative of the metazoans [23].

The nucleotide coding sequences (CDS) for ADF and CFL proteins from each species were acquired through NCBI (www.ncbi.nlm.gov) and TAIR (www.arabidopsis.org) using the online protein databases. BLASTp was used to verify that all ADF/CFL variants had been obtained for each species. Accession numbers are listed in S1 and S2 Tables. The coding sequence of the ADF/CFL variants were translated to amino acid sequences for alignment. Sequences were aligned using ClustalW [24] within MEGA 5.05 [25]. The BLOSUM protein weight matrix was used for aligning the sequences; once aligned, the translated sequences were converted back to nucleotide sequences and the alignment was manually adjusted using Se-Al v2.0a11. Any fully redundant or nearly identical protein variants (i.e., < 5 nucleotide differences) were excluded from the analysis. While there is data on function for many of the protein variants used for our analyses, this is not true for all of the proteins included. To avoid the inclusion of non-functional proteins, we only included those ADF/CFL protein variants that did not contain stop codons or frame shift mutations.

### Phylogenetic Analyses

Phylogenetic analyses were performed separately on the plant ADF and animal ADF/CFL gene family. The program MrModelTest [26] was used to determine the best-fit substitution model for each dataset, and Bayesian phylogenetic analyses were conducted using the program MrBayes 3.1 [27]. For the plant ADF dataset, we used the general-time-reversible (GTR) model of nucleotide substitution [28, 29] with gamma distributed rate variation. Two independent runs were implemented for 10,000,000 generations, each with a tree sample frequency of every 100 generations. For the animal ADF/CFL dataset, we used the GTR model of nucleotide substitution with gamma distributed rate variation and allowing for a proportion of invariable sites. We completed two independent runs of 5,000,000 generations, each with a sample frequency of every 100 generations. For each analysis the consensus tree was compiled after excluding the first 25% of sampled trees as burnin. Maximum-likelihood and neighbor-joining phylogenetic analyses were also performed in Mega 5.05 [25] and the results were congruent with Bayesian inferences.

### Selection Analyses

To investigate the selective pressure across the ADF/CFL gene phylogenies, we compared the rate of nonsynonymous (dN) to synonymous (dS) mutations both across the ADF/CFL coding sequences as well as across the phylogeny. This ratio, commonly referred to as dN/dS or ω, indicates the type and strength of selection acting on a protein. A rate of nonsynonymous mutations less than the rate of synonymous mutations (ω < 1) is a signature of negative/purifying selection (i.e., conserved residues). If a protein is evolving neutrally, then the rate of nonsynonymous mutation is equal to the rate of synonymous mutations (ω = 1). Positive/diversifying selection (i.e., residues with accelerated rates of evolution) leaves a signature of a higher rate of nonsynonymous mutations than synonymous mutations (ω > 1). The main goal of this paper is not to test whether gene family members have evolved through positive selection, but rather to identify protein variants and/or regions and residues within the protein that have experienced accelerated rates of evolution (i.e., ω > 1). Thus, we utilized the program fitModeL [30] to estimate the degree of selective pressure exerted on the ADF/CFL protein variants. fitModeL allows for selection to vary across phylogenetic lineages as well as between amino acid sites without making *a priori* assignments [31]. A series of nested models of codon evolution were tested to determine the best-fit model for the plant ADF and animal ADF/CFL datasets separately. In total, four models of codon evolution were tested: M0, M3, M3+S1, and M3+S2. The M0 model is the null model and assumes a single ω value for every amino acid position across all phylogenetic lineages. This null model was compared to the discrete model of codon evolution (M3), which allows selective pressure to vary across the protein sequence. The M3 model allows for three selection rate categories (ω_1_, ω_2_, and ω_3_) with the only restriction being that ω_1_ < ω_2_ < ω_3_ [31]. Even though the M3 model of codon evolution allows for selection to vary across the protein sequence, the estimates are held constant across phylogenetic lineages. The next two models (M3+S1 and M3+S2) allow for selection to vary across phylogenetic lineages. For both the M3+S1 and M3+S2 models, each codon position for all proteins in the phylogeny is assigned a probability of being in a particular rate category (ω_1_ vs. ω_2_ vs. ω_3_). The M3+S1 model assumes an unbiased switching between rate categories across phylogenetic lineages for a particular codon position. The M3+S2 model tests the added parameter of unequal switching between rate categories, allowing for certain sites to switch more frequently from one selection rate category (e.g., ω_1_) to another selection rate category (e.g., ω_3_). A series of nested log-likelihood ratio tests (LRT) were used to determine the best-fit model for each dataset.

Using the fitModeL results, the nodes and protein variants that had the highest probability of being in the ω_3_ rate category were identified and recorded for each amino acid position. This process was repeated for the ω_1_ and ω_2_ rate categories. This was plotted along the aligned codon positions to identify those sites that had the most protein and nodes within a phylogeny in the ω_3_ rate category. For each one of these sites of interest, each branch in the phylogeny was assigned to the ω category in which it had the highest probability of belonging to based on the fitModeL analyses.

Three-dimensional protein modeling was performed using a protein data bank (pdb) template identification and 3D modeling programs in Swiss-Model Workspace (swissmodel.expasy.org). The Arabidopsis ADF1 pdb structure 1f7sA was used for modeling *Zea mays* ADF5, human CFL1 3josW for itself, and chicken cofilin 1tvjA for *Danio rerio* CFL2. A few residues at the N- and C-terminal ends of Arabidopsis ADF11 and Arabidopsis ADF6 were too divergent to be mapped to the best template structures. Structures were visualized in pymol (www.pymol.org). Amino acids of interest were first identified in the aligned sequences used for analysis and then located and highlighted in the sequences used for modeling.

## Results

### Sequence Acquisition and Alignment

In total, 60 plant ADF protein variants from 8 plant species and 24 ADF/CFL protein variants from 12 animal species (Table 1) were included in our study. The *S*. *moellendorffii* genome is comprised of two haplotypes that are nearly identical in nucleotide sequence [32], and initially four ADF protein variants were identified from this genome: Sm146459, Sm230142, Sm270871, and Sm233521. Sm270871 and Sm233521 are nearly identical to Sm148459 and Sm230142, respectively, differing exclusively in synonymous nucleotide changes. These two pairs of ADF protein variants are most likely a product of the two genomic haplotypes; therefore, only one pair of ADF sequences was used for analysis (Sm146459 and Sm230142). Both protein variants were expressed, with Sm146459 being expressed at 2-fold higher levels than Sm230142 in the frond tissue examined (S1 Fig).

**Table 1:** The number of ADF/CFL protein variants used for each species analyzed.

	Species	# of Variants		Species	# of Variants
	*Physcomitrella patens*	1		*Monosiga brevicolis*	1
	*Selaginella moellendorffii*	2		*Ciona intestinalis*	1
	*Zea mays*	5		*Branchiostoma floridae*	1
	*Oryza sativa (japonica)*	9		*Drosophila melanogaster*	1
	*Mimulus guttatus*	11		*Caenorhabditis elegans*	1
**Plant ADF**	*Vitis vinifera*	7	**Animal CFL/ADF**	*Salmo salar*	4
	*Populus trichocarpa*	14		*Danio rerio*	4
	*Arabidopsis thaliana*	11		*Xenopus tropicalis*	2
				*Gallus gallus*	2
				*Sus scrofa*	3
				*Mus musculus*	3
				*Homo sapiens*	3

For the animal dataset, each of the four invertebrate species included in the analysis contained only a single ADF/CFL sequence. The only exception was amphioxus (*Branchiostoma floridae*), which had multiple annotated ADF/CFL sequences. Given that all the amphioxus sequences grouped closely together in a preliminary phylogenetic analysis (data not shown), only a single variant was included in the analysis. Of the vertebrates, the two fish species (*S*. *salar* and *D*. *rerio*) and mammalian species possess the most variants with 3 CFL variants each, while the amphibian and bird species possess only two CFL/ADF variants (Table 1).

### Phylogenetic Analyses

**Fig 1:**
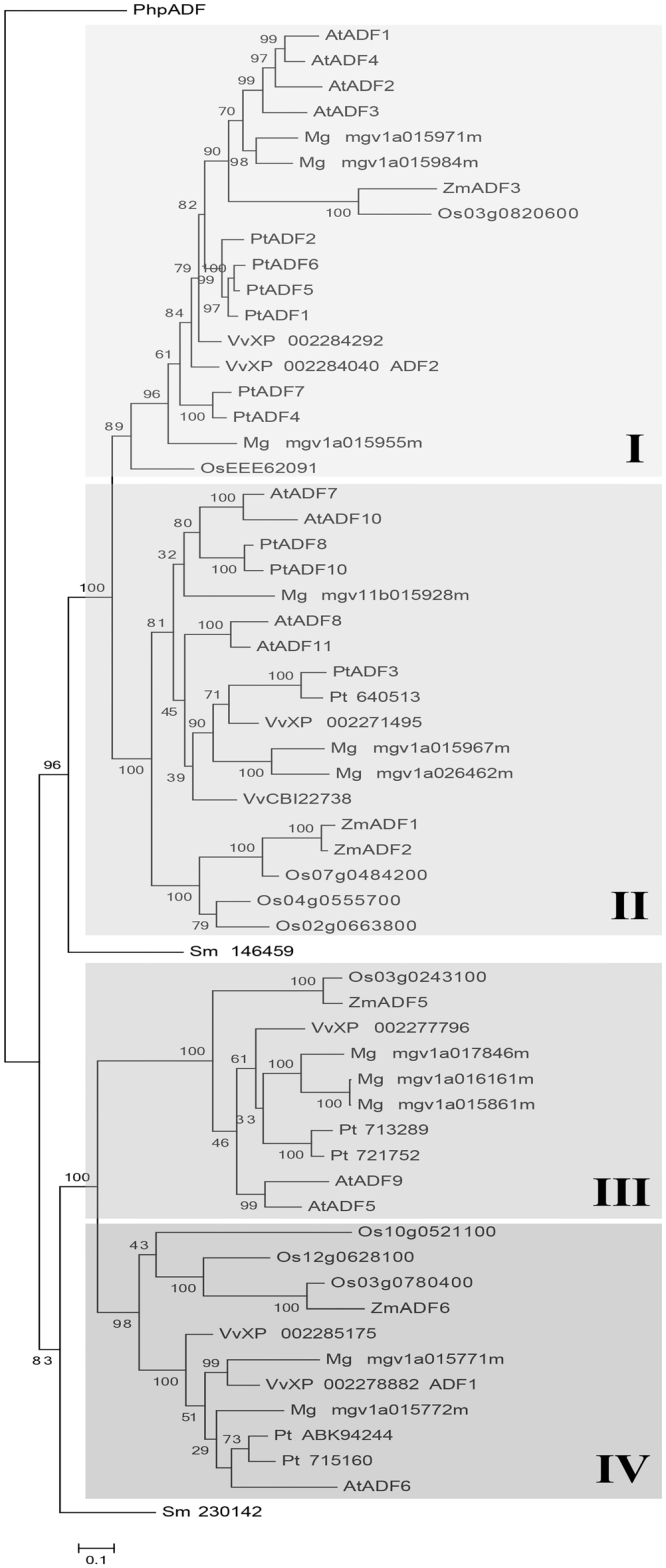
Bayesian phylogenetic analysis of the plant ADF gene family. The four ADF subclasses are highlighted in boxes. Species designations are as follows: At, *Arabidopsis thaliana*; Pt, *Populus trichocarpa*; Mg, *Mimulus guttatus*; Vv, *Vitis vinifera*; Os, *Oryza sativa*; Zm, *Zea mays*; Sm, *Selaginella moellendorffii*; and Php, *Physcomitrella patens*. ADF family number is given next to species designation when available. For those sequences that did not have a published family number on NCBI, the accession number is given. Accession numbers for all plant ADF variants used in analyses can be found in S1 Table.

The phylogenetic analysis of the plant ADF variants recovered the four ancient subclasses previously described (Fig 1) [11, 12]. Of note in our analysis were the placement of the moss *P*. *patens* and lycophyte *S*. *moellendorffii* ADF variants (Fig 1). The *P*. *patens* ADF variant grouped outside of the four subclasses, while one *S*. *moellendorffii* ADF variant (Sm146459) was basal to subclasses I and II and the other *S*. *moellendorffii* ADF variant (Sm230142) was basal to subclasses III and IV (Fig 1). The two most diverse subclasses were subclasses I and II, with more ADF variants per species being represented in these two subclasses. Even though subclass III and subclass IV contain fewer ADF variants per species, each angiosperm species still had at least one ADF variant within these two subclasses (Fig 1). Finally, the phylogeny indicates that within subclasses II, III, and IV the monocot ADF variants form a distinct group from dicot ADF variants. This division between monocot and dicot ADF variants was not seen in subclass I.

**Fig 2:**
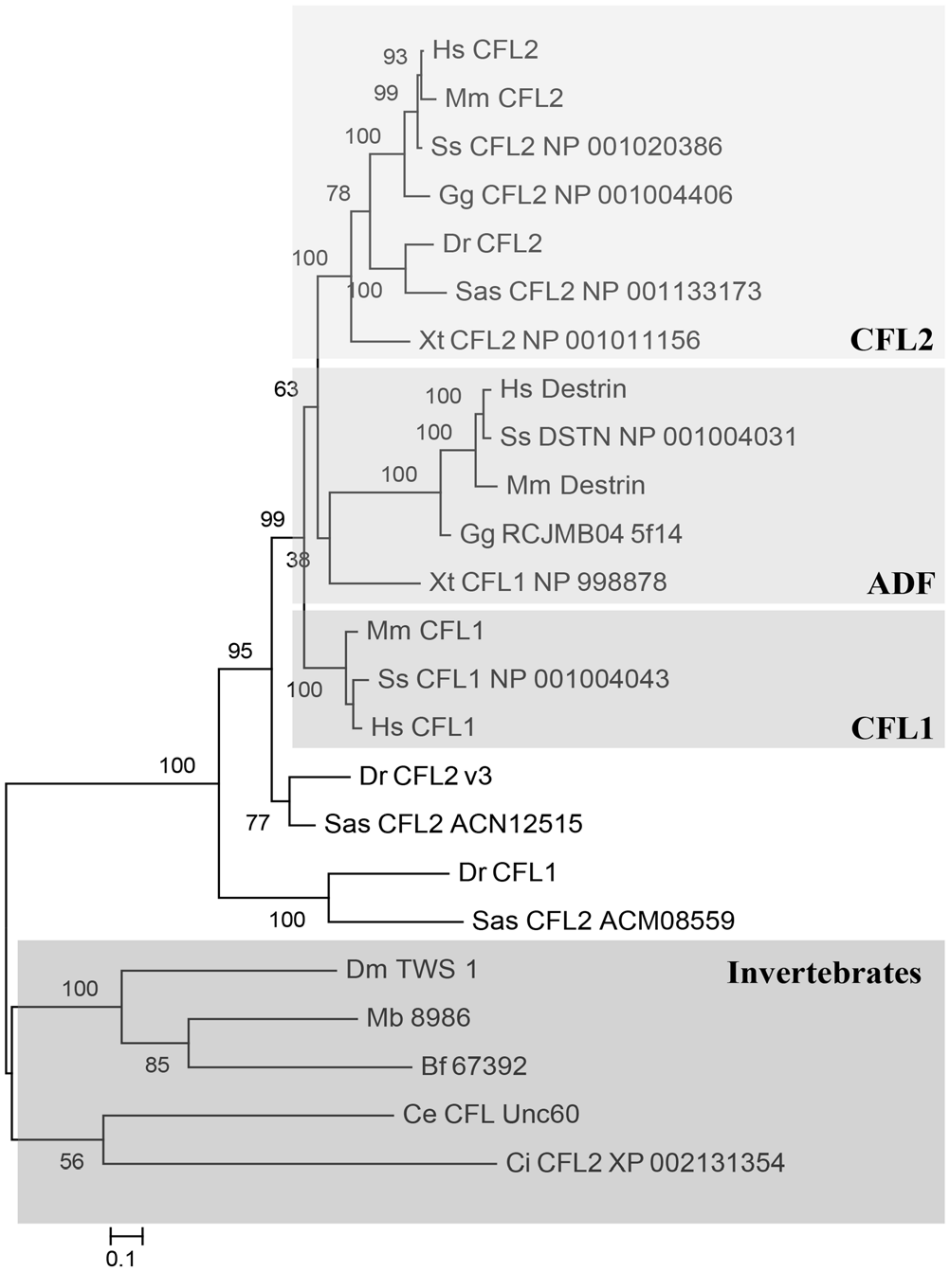
Bayesian phylogenetic analysis of the animal ADF/CFL gene family. The three ADF/CFL classes are highlighted in boxes. Species designation are as follows: Hs, *Homo sapiens*; Mm, *Mus musculus*; Ss, *Sus scrofa*; Gg, *Gallus gallus*; Xt, *Xenopus tropicalis*; Dr, *Danio rerio*; Sas, *Salmo salar*; Bf, *Branchiostoma floridae*; Ci, *Ciona intestinalis*; Dm, *Drosophila melanogaster*; Ce, *Caenorhabditis elegans*; Mb, *Monosiga brevicollis*. ADF/CFL family number is given next to species designation when available. For those sequences that did not have a published family number on NCBI, the accession number is given. Accession numbers for all animal ADF/CFL variants used in analyses can be found in S2 Table.

The phylogenetic analysis of the animal ADF/CFLs revealed that the invertebrate and vertebrate ADF/CFL sequences group separately from one another (Fig 2). It should be noted that the chordate species (*C*. *intestinalis* and *B*. *floridae*) did not form a monophyletic group in the phylogeny. The vertebrate ADF/CFL variants further diverge into three main classes: CFL1, CFL2, and ADF/Destrin. Of these, only mammals contained representatives in all three classes, and only in the mammalian lineage did we find the non-muscle CFL1 variants. All other vertebrate species had representatives only in the CFL2 and ADF/Destrin classes. Our phylogenetic analysis provided greater resolution for two particular lineages than has been previously published for the amphibian *X*. *tropicalis* and the two bony fish species, *D*. *rerio* and *S*. *salar*. As with mammals, fish possess three distinct ADF/CFL variants. However, only the *S*. *salar* and *D*. *rerio* CFL2 variant grouped within the other vertebrate ADF/CFL sequences, the muscle-specific CFL2 class. The remaining fish ADF/CFL variants grouped outside of the three vertebrate classes, possibly as a fourth class of ADF/CFL variants, suggesting that these protein variants have progressed on an independent evolutionary trajectory that is unique to fishes (Fig 2). The frog *Xenopus tropicalis* has two ADF/CFL variants that have historically been shown to group with each other, outside of the three conserved classes of vertebrate ADF/CFLs [8, 9]. In our analysis, the two *X*. *tropicalis* variants grouped within the vertebrate sequences, with one variant in the vertebrate ADF/Destrin group and the second variant in the muscle-specific CFL2 group (Fig 2).

### Selection Analyses

We tested whether selective constraint varies across the ADF/CFL plant and animal gene families. Focusing on the plant ADF gene family, the M3+S2 model of codon evolution best fit the data, suggesting that selection varies both across the protein sequence as well as across the gene phylogeny (Table 2). The results from this analysis revealed that while most codons were under tight selective constraint, there were rare instances of positive selection. Sixty-four percent of codon positions were found in the ω_1_ rate category with an estimate of ω_1_ = 0.01, indicating very strong purifying selection, while 0.9% of sites were found to be under positive selection (ω_3_ = 19.99, Table 2).

**Table 2:** Selection across the plant ADF and animal ADF/CFL gene family.

	Model	ln(Likelihood)	Comparison	LRT Statistic	df	p-value	ω Estimates (Proportion of sites)
	M0	-13191.73					ω1 = 0.07(1.0)
**Plant ADF**	M3	-12916.57	M0 vs M3	550	4	p<0.0001	ω1 = 0.02(0.48); ω2 = 0.12(0.38); ω3 = 0.26(0.14)
	M3	-12916.57					
	M3S1	-12772.53	M3 vs M3S1	288	1	p<0.0001	ω1 = 0.007(0.64); ω2 = 0.15(0.30); ω3 = 1.15(0.06)
	M3S2	-12737.16	**M3S1 vs M3S2**	70.74	2	**p<0.0001**	ω1 = 0.010(0.64); ω2 = 0.13(0.35); **ω3 = 19.999(0.009)**
							R(1,2) = 0.17; R(1,3) = 0.001; R(2,3) = 12.31
	M0	-7809.34					ω1 = 0.03(1.0)
**Animal ADF/CFL**	M3	-7617.07	M0 vs M3	384	4	p<0.0001	ω1 = 0.005(0.5); ω2 = 0.025(0.37); ω3 = 0.21(0.13)
	M3	-7617.07					
	M3S1	-7496.88	M3 vs M3S1	240	1	p<0.0001	ω1 = 0.00001(0.66); ω2 = 0.03(0.22); ω3 = 0.40(0.12)
	M3S2	-7473.81	**M3S1 vs M3S2**	46.14	2	**p<0.0001**	ω1 = 0.003(0.65); ω2 = 0.05(0.33); **ω3 = 19.53(0.015)**
							R(1,2) = 0.06; R(1,3) = 0.001; R(2,3) = 3.91

A similar pattern of codon evolution was seen for the animal CFL/ADF gene family, as the M3+S2 model of codon evolution was also shown to be the best fit (Table 2). The animal CFL/ADF protein variants were generally under tight selective constraints (ω_1_ = 0.01, *p*_1_ = 0.65); however, as with the plant ADFs, there were rare episodes of significant accelerated rates of evolution with a ω_3_ estimate of 19.53 (*p*_3_ = 0.015, Table 2). For both the plant and animal ADF/CFL proteins, there was a more switching from the ω_2_ to the ω_3_ selection category than between other pairs of categories. We note that the estimate of ω_2_ was considerably lower than 1 in our analyses, which suggests that sites were switching from a strong constraint to positive selection (e.g., for plant ADFs, switching occurs from an ω_2_ of 0.13 to an ω_3_ of 19.99, Table 2).

**Fig 3:**
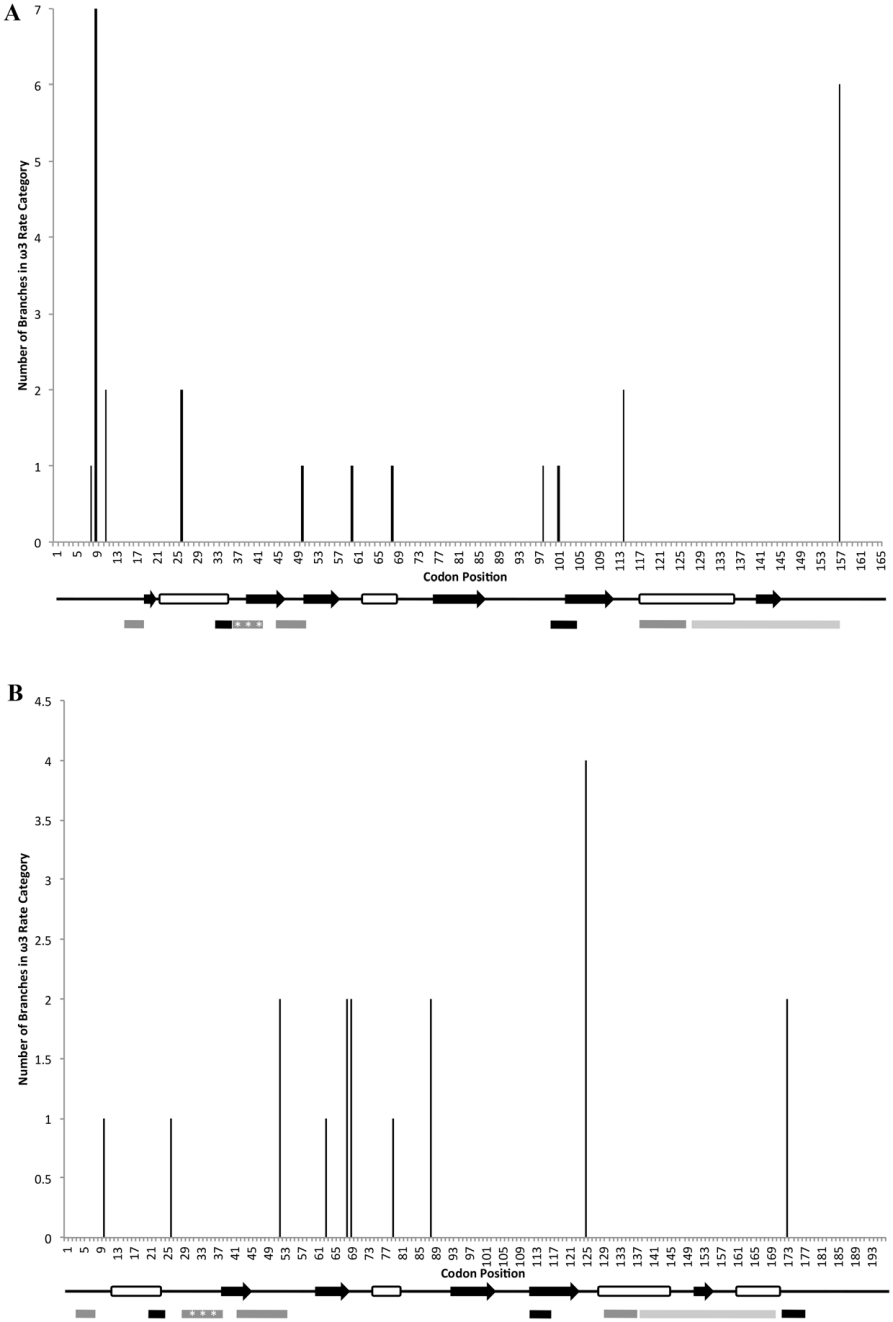
The number of branches with the highest probability of being in the ω3 rate category plotted across each codon position for the (A) plant ADF and (B) animal CFL/ADF protein variants. A linear representation of the secondary structure is depicted directly below each graph, where open cylinders represent α-helices and black arrows represent β-sheets. Conserved binding regions are depicted below the secondary structures, with black bars represent F-actin binding regions, dark gray bars represent regions that are involved in both G-actin and F-actin binding, light gray bars are regions involved in only G-actin binding, and the dark gray bar with asterisks is the nuclear localization sequence. Tertiary structure and binding domains are based on [33] and [34]. The numbering of codon positions is based on protein sequence alignments used for phylogenetic analyses and selection analyses.

**Fig 4:**
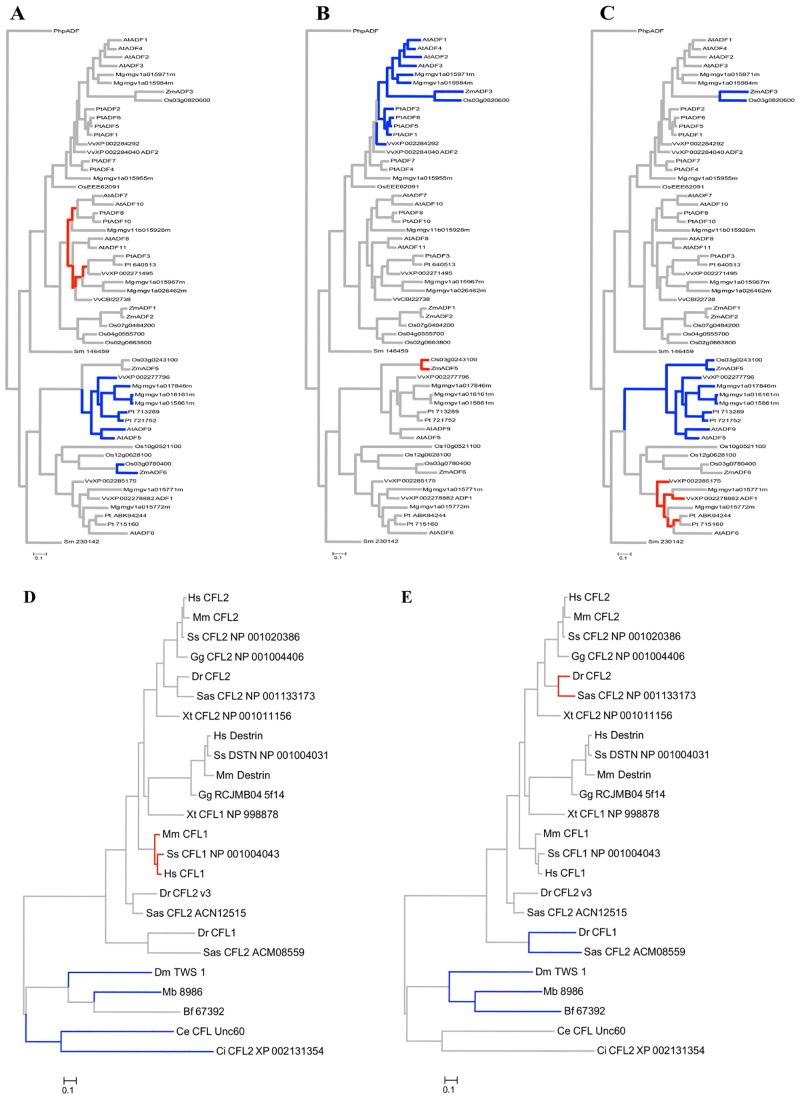
Codon positions with highest probability of being in the ω_3_ rate category within plant and animal phylogenies. The codon positions (A) 157 (B) 26 and (C) 9 were found to be in the ω_3_ rate category in the plant ADF phylogeny. The codon positions (D) 125 and (E) 69 were found in the ω_3_ rate category in the animal ADF/CFL phylogeny. Red branches have the highest probability of being in the ω_3_ rate category, gray branches represents branches with the highest probability in the ω_2_ rate category, and blue branches represents branches with the highest probability of being in the ω_1_ rate category.

**Fig 5:**
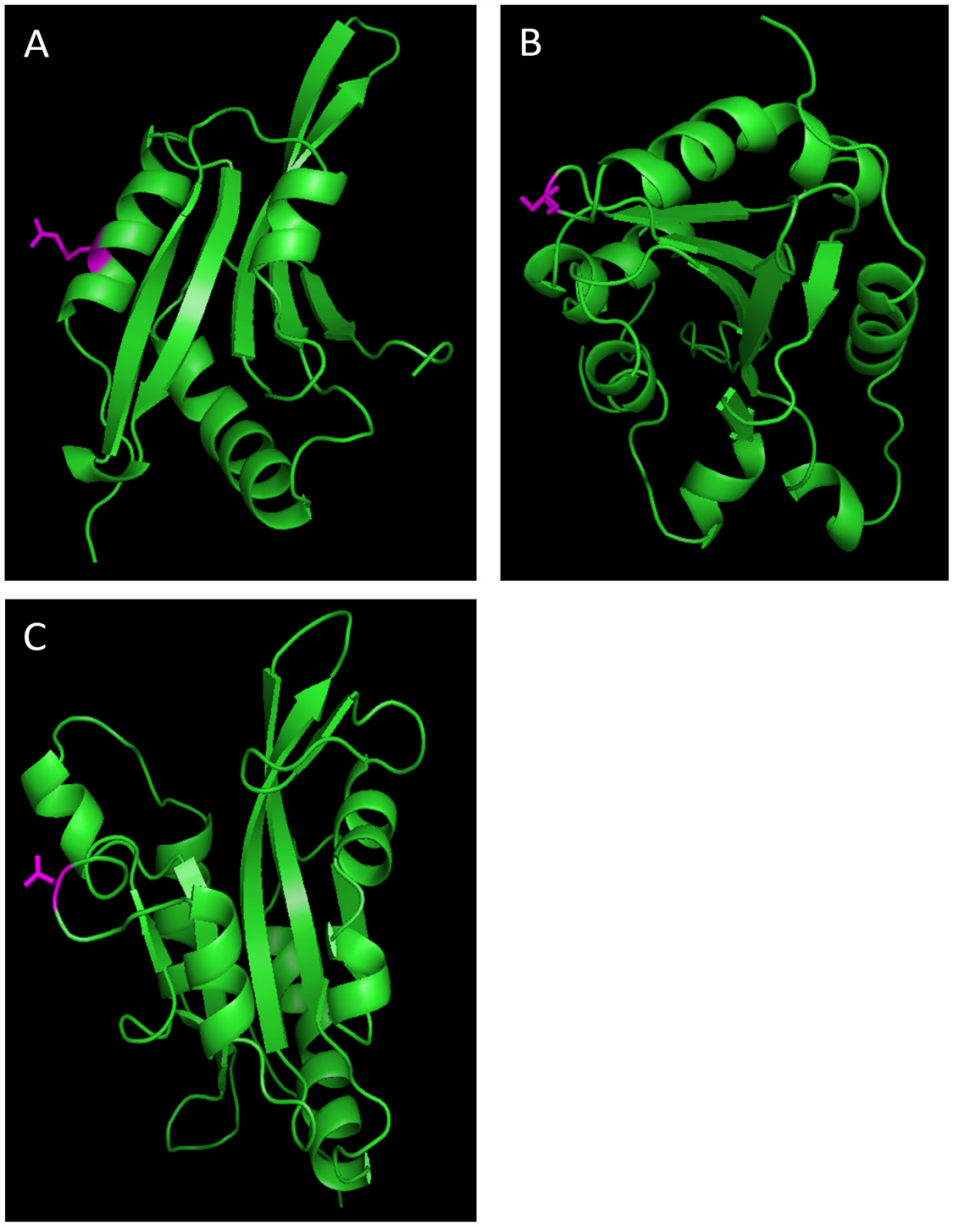
Three dimensional protein models of ADF/CFL proteins highlighting sites with accelerated rates of evolution (ω_3_ rate category) in pink. (A) *Z*. *mays* ADF5 proteins with codon position 26 highlighted. (B) *H*. *sapiens* CFL1 protein with codon position 125 highlighted. (**C**) *D*. *rerio* CFL2 protein with codon position 69 highlighted.

To discern where along the ADF/CFL protein sequence these sites occurred, the number of branches within the phylogeny that had the highest probability of containing a particular amino acid position within the ω_3_ rate category (i.e., the highest rate of dN/dS) was plotted across the linear protein sequence (Fig 3A and 3B). All codon positions are based on the protein alignments used for the phylogenetic analysis and selection analyses. In the plant ADF gene family, 10 of the 11 amino acid positions with highest probability of being grouped within the ω_3_ rate class were located outside of any known G-actin and F-actin binding domains and were also not within the nuclear localization sequence (NLS) (Fig 3A). Two of the sites, codon positions 9 and 157, had a relatively large number of branches in the ω_3_ rate class. Plotting these branches on the phylogeny identified subclass specific as well as monocot and dicot specific patterns of relaxed purifying selection (Fig 4A–4C). Codon position 9 is located in the N-terminal tail of the ADF protein and was found to have the highest probability of being in the ω_3_ rate category for dicot ADF variants within subclass IV only (Fig 4C). Likewise, codon position 157 is located in the C-terminal tail of the ADF protein and was found to be in the ω_3_ rate category for Subclass II dicot ADF variants only (Fig 4A). Additionally, codon position 26 was found to have experienced accelerated rates of evolution in the subclass III monocot ADF variants (Fig 4B). Mapping codon position 26 onto a 3D model of *Z*. *mays* ADF5, a subclass III monocot ADF variant, showed that this particular site is located on an alpha helix that lies on the exterior of the protein with its side chain exposed (Fig 5A).

For the animal CFL/ADF protein variants, eight of the 10 sites with the highest probability of being in the ω_3_ rate category were located outside of known actin-binding and NLS sequences as well as outside of the α helices and β sheets (Fig 3B). The two exceptions were codon position 63, which is located within a β sheet, and codon position 173, which is located within a conserved F-actin binding domain. Codon position 63 was in the ω_3_ rate category for a single *S*. *salar* protein variant, while codon 173 had the highest probability of being in the ω_3_ rate category for the branch leading to the fish specific CFL group and a single *S*. *salar* sequence within that group (data not shown). Plotting the remaining branches that had amino acid positions within the ω_3_ rate category across the animal phylogeny showed some interesting patterns of codon evolution. Codon position 125 was found to have the highest probability of experiencing accelerated rates of evolution for the mammalian CFL1 protein variants (Fig 4D), and codon position 69 was in the ω_3_ rate category for the *S*. *salar* and *D*. *rerio* protein variants that cluster with the CFL2 protein variants (Fig 4E). Interestingly, this later site was in the ω_1_ rate category for the two *S*. *salar* and *D*. *rerio* protein variants that are basal to the rest of the CFL/ADF protein variants (Fig 4E). When codon position 125 was mapped onto the 3D structure of the *H*. *sapiens* CFL1 protein variant this showed that this site lies on an outer loop of the protein towards the C-terminal end (Fig 5B). Likewise, when codon position 69 was mapped onto a 3D model of *D*. *rerio* CFL2, it was shown to also lie on an unstructured loop on the surface of the protein and not in a domain with known function (Fig 5C).

## Discussion

Gene duplication is a primary mechanism for the evolution of new genes and functions [35]. Initially, the ADF/CFL proteins were thought to be involved solely in the depolymerization of actin filaments [1, 4]. Indeed, mammalian cytoplasmic actin variants and some protist actins immediately basal to animals, plants, and fungi can fully complement all the extreme developmental defects of plant vegetative actin null mutants, suggesting that most of actin’s interactions with its binding partners are extremely ancient and conserved [36, 37]. However, it is now clear that animal and plant ADF/CFL variants are involved in many more recently evolved cellular processes and are essential to cellular development [4]. Thus, while most ancient functions in the cytoplasmic actin system have been conserved, gene duplication likely facilitated the diversity and subsequent versatility of actin and actin-binding proteins.

Our revised phylogenetic analyses provide insights into the evolutionary history and expansion of this important family of proteins. The inclusion of the two basal plant species, the bryophyte *P*. *patens* and the lycophyte *S*. *moellendorffii*, improves our understanding of the divergence of the four plant ADF subclasses. The placement of these ADF variants within the phylogeny (Fig 1) suggests an ancient split occurred in the plant ADF gene family after common ancestry with moss 600 mya, but before the common ancestor with lycophytes 450 mya (S1 Table). The updated plant ADF phylogeny also revealed that there has been gene duplication and divergence among monocot and dicot ADF variants within three of the subclasses (II, III, and IV). This has been suggested in previous studies [11], but our analysis provides strong evidence for this pattern. Interestingly, we find dicot and monocot specific patterns of codon evolution within these same three subclasses (discussed below). In contrast, subclass I did not have detectable divergence between the monocot and dicot ADF variants.

In the animal ADF/CFL phylogeny, we find a major split between the invertebrate and vertebrate ADF/CFL variants, as has also been noted in other studies [8, 9]. While most invertebrates possess a single ADF/CFL protein variant, there are three classes of ADF/CFL variants within vertebrates (CFL1, CFL2, and ADF/Destrin) [8, 9]. We find that only mammals have a representative in all three vertebrate ADF/CFL classes, while other vertebrates (birds and amphibians) have only CFL2 and ADF/Destrin representatives. The two fish species included in this study (zebrafish and salmon) contain three ADF/CFL variants, but only one of their variants (CFL2) grouped within one of the three conserved mammalian classes; the other two formed a fish-specific clade. Also of note is the resolution our phylogeny provides for the *X*. *tropicalis* CFL/ADF sequences. Previous analyses had the two well-characterized *Xenopus* protein variants forming their own phylogenetic group outside of the three known vertebrate ADF/CFL classes, resembling the fish ADF/CFL variants [8, 9]. Our analysis resolves their placement to within the phylogeny, with one variant in the ADF/Destrin class and the second in the CFL2 class.

We found that the ADF/CFL proteins have been under tight selective constraint for the majority of their evolutionary history, which is not surprising as these proteins are small and are mainly composed of conserved actin binding domains. However, we also identify the presence of rare, episodic events of positive selection. For both the plant and animal phylogenies, about 1–2% of the amino acids have experienced high rates of evolution (ω_3_ > 19). The switching pattern reveals that these sites are not simply switching from neutral to relaxed selection, but rather between strong selective constraints and strong diversifying selection. Most of the codons with a high probability of being in the ω_3_ rate category were located outside of any known conserved binding regions or conserved secondary structures, indicating that there is not any particular binding domain with its function known at this time that can be implicated in functional diversification (Fig 3). There were two exceptions within the vertebrate ADF/CFL proteins, codon positions 63 and 173, but neither site showed any clear pattern except that they were specific to two unrelated fish ADF/CFL variants.

We suggest that all of the amino acid positions with accelerated rates of evolution, both in plants and animals, were located in regions of the protein that could serve in new functional roles. Within the plant ADF protein variants, codon position 157 had an accelerated rate of evolution only in subclass II dicot ADF protein variants; this site is just outside of the conserved G-actin binding domain of the C-terminus. Codon position 9 was in the ω_3_ rate category only in the dicot variants in subclass IV; this site is located within a functional region of the protein that is unique to subclass IV variants, which is an extended N-terminus region not found in any other ADF subclass. While codon position 9 had an accelerated rate of evolution in subclass IV, it was found to be in the ω_1_ rate category for the subclass III ADF variants suggesting that this region/site may have had a role in the divergence of subclass III and subclass IV dicot ADF variants. Finally, codon position 26 was in the ω_3_ rate category for the monocot variants in subclass III; interestingly, this site is within a known α-helix of the ADF-H domain, which is on the external portion of the protein with its side chain exposed to the cytoplasmic milieu. Based on the position of amino acid 26 in *Z*. *mays*, this site could be important in forming new binding interactions, or possibly serve as a new target for protein regulation. ADF/CFL proteins are known to bind to multiple different partners in addition to actin, and are also known to have site-specific modifications (e.g., serine phosphorylation) that regulate their multifaceted role within the eukaryotic cell. This particular site is thus a candidate for the establishment of a new protein interaction or new level of regulation for the monocot subclass III ADFs.

In the animal ADF/CFL gene family codon 125 and codon 69 showed an accelerated rate of evolution. Codon 125 does not occur in any conserved binding or structural regions and had an accelerated rate of evolution only in mammal-specific CFL1 class. This codon position mapped to an outer loop of the *H*. *sapiens* protein model, suggesting that it could be involved in new protein interactions. Codon position 69 may represent a similar case as it maps to an outer loop of the fish CFL2 protein structure, and is evolving rapidly only in the CFL2 fish protein variants. In *D*. *rerio* CFL2 is expressed solely in the muscle tissue while the CFL1 protein variant is an essential component of embryonic tissue during gastrulation [38]. Even though this site is relatively constrained across nearly all vertebrate ADF/CFL variants (ω_2_ = 0.05), it is particularly constrained in the two basal fish CFL1 variants (ω_1_ = 0.003). We suggest that this site may have played a role in the divergence between two classes of fish ADF/CFL variants.

In summary, our results provide clues to the evolutionary processes and mutations that may have facilitated functional divergence of gene family members. Our analyses show that ADF/CFL proteins have been under very strong purifying selection, but that selective pressure has varied during key points in the evolutionary history of the ADF/CFL proteins that could have implications in functional divergence. The locations of four amino acid residues with accelerated rates of evolution (amino acids 9 and 26 in plants and amino acids 125 and 69 in animals) on the protein’s exterior suggests these sites may have important functional implications and are targets for future molecular genetic dissections. It is our hope that the results from our study will enable future studies on the molecular characterization of the members of the ADF/CFL gene and protein variants.

## Supporting Information

S1 Fig RNA levels of two *S*. *moellendorffii* ADF variants, Sm 146459 (Sm146) and Sm 230142 (Sm230). Total RNA was extracted from leaf tissue. Actin 1 was used as an endogenous control and all qRT-PCR primers were designed from confirmed sequences. Expression was calculated using the dCT method.
(TIF)

S1 Table Plant species sampled for ADF/CFL sequences and their divergence times from a common ancestor with Arabidopsis. Estimates of divergence times among species were extrapolated from references [39, 40, 41, 42]. Accession numbers are given for all sequences used in analyses.
(DOCX)

S2 Table Animal species sampled for ADF/CFL sequences and their divergence times from a common ancestor with *H*. *sapiens*. Estimates of divergence times among species were extrapolated from references [23, 43]. Accession numbers are given for all sequences used in analyses.
(DOCX)
