# Supplementary figures and images for: Phylogenetic Patterns of Codon Evolution in the ACTIN-DEPOLYMERIZING FACTOR/COFILIN (ADF/CFL) Gene Family
Source: PLoS One. 2015 Dec 30;10(12):e0145917. doi: 10.1371/journal.pone.0145917 (PMC4696841; doi:10.1371/journal.pone.0145917)

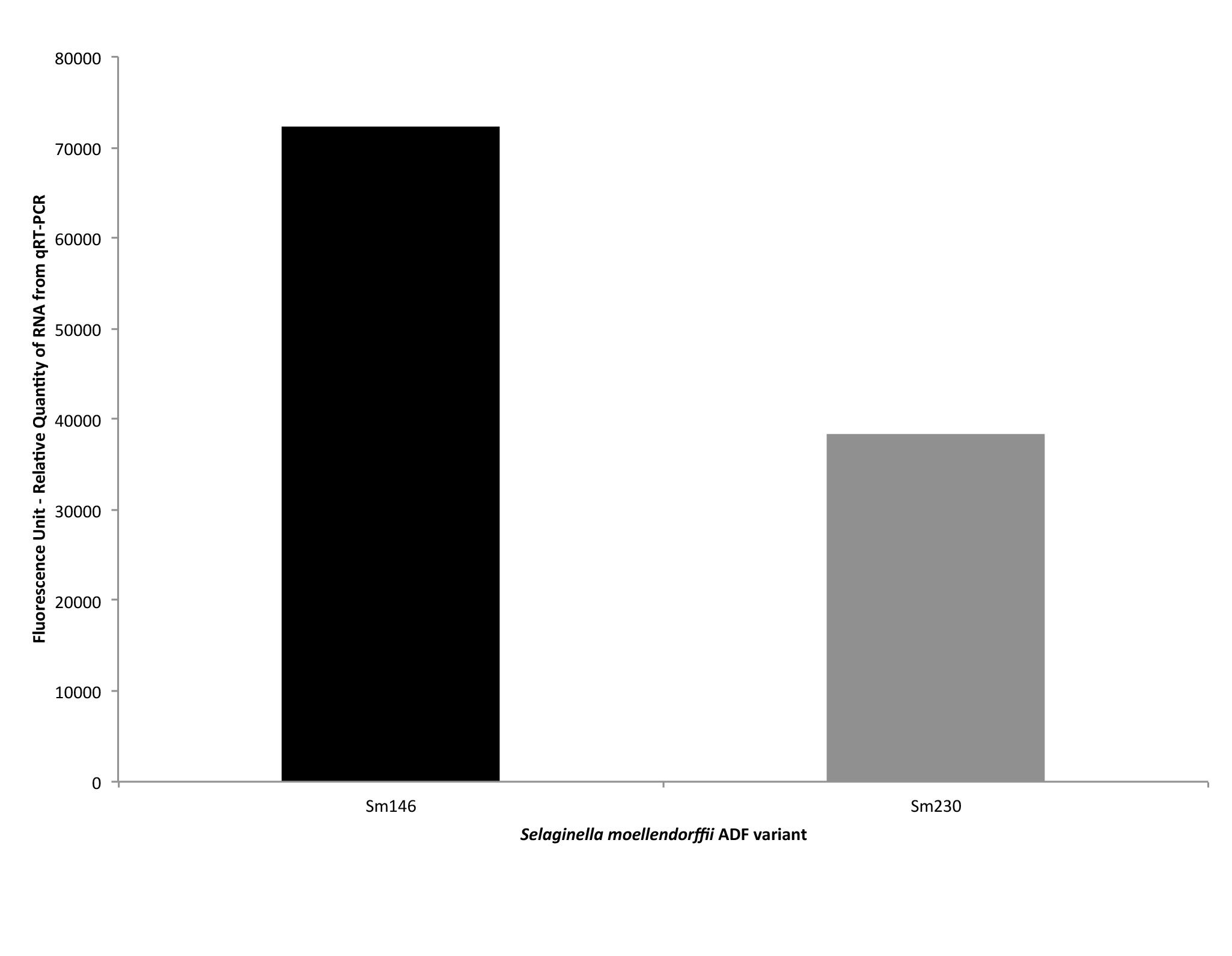

Supplement: S1 Fig — Total RNA was extracted from leaf tissue. Actin 1 was used as an endogenous control and all qRT-PCR primers were designed from confirmed sequences. Expression was calculated using the dCT method. (TIF) [file pone.0145917.s001.tif]
