# Supplementary material for: Phylogenetic Patterns of Codon Evolution in the ACTIN-DEPOLYMERIZING FACTOR/COFILIN (ADF/CFL) Gene Family
Source: PLoS One. 2015 Dec 30;10(12):e0145917. doi: 10.1371/journal.pone.0145917 (PMC4696841; doi:10.1371/journal.pone.0145917)
Supplement: S1 Table — Estimates of divergence times among species were extrapolated from references [39, 40, 41, 42]. Accession numbers are given for all sequences used in analyses. (DOCX) [file pone.0145917.s002.docx]

**Supplementary Table 1** Plant species sampled for ADF/CFL sequences and their divergence times from a common ancestor with Arabidopsis

| **Species** | **Informative Classification** | **Estimated Divergence Times** | **Ascension #** |
| --- | --- | --- | --- |
| *Physcomitrella patens* | Non-vascular plant, Bryophyta | 600 mya | XP_001777224 |
| *Selaginella moellendorffii* | Vascular plant, Lycopodiophyta | 450 mya | XP_002963013 XP_002969368 |
| *Zea mays* | Angiosperm, monocot,Poaceae | 250 mya | ACG46218 NP_001105474 ACG37280 NP_001148661 NP_001105590 |
| *Oryza sativa japonica* (rice) | Angiosperm, monocot, Poaceae | 250 mya | Os12g0628100 Os04g0555700 Os03g0820600 Os03g0780400 Os03g0243100 Os02g0663800 EEE62091 Os10g0521100 Os07g0484200 |
| *Vitis vinifera* | Angiosperm, dicot, Vitaceae | 110 mya | XP_002278882 XP_002284040 CBI22738 XP_002271495 XP_002284292 XP_002285175 XP_002277796 |
| *Mimulus guttatus* | Angiosperm, dicot, Scrophulariaceae | 110 mya | mgv1a015772m mgv1a015771m mgv1a015861m mgv1a015971m mgv1a015984m mgv1a015967m mgv1a026462m mgv1a016161m mgv1a017846m mgv11b015928m |
| *Populus trichocarpa* | Angiosperm, dicot, Salicaceae | 70 mya | XP_002299887 XP_002299888 XP_002303579 XP_002311154 XP_002314194 XP_002314195 XP_002316301 XP_002322471 XP_002318237 XP_002313717 XP_002305510 XP_002298043 XP_002307641 XP_002300779 |
| *Arabidopsis thaliana* | Angiosperm, dicot, Brassicaceae | 0 mya | NP_190187 NP_566882 NP_851227 NP_851228 NP_565390 NP_565719 NP_194289 NP_567182 NP_195223 NP_568769 NP_171680 |

Estimates of divergence times among species were extrapolated from the following references: Heckman et al., 2001; Yoon et al., 2004; Moore et al., 2007; Zimmer et al., 2007.
