# Supplementary material for: Phylogenetic Patterns of Codon Evolution in the ACTIN-DEPOLYMERIZING FACTOR/COFILIN (ADF/CFL) Gene Family
Source: PLoS One. 2015 Dec 30;10(12):e0145917. doi: 10.1371/journal.pone.0145917 (PMC4696841; doi:10.1371/journal.pone.0145917)
Supplement: S2 Table — Estimates of divergence times among species were extrapolated from references [23, 43]. Accession numbers are given for all sequences used in analyses. (DOCX) [file pone.0145917.s003.docx]

**SI Table 2** Animal species sampled for ADF/CFL sequences and their divergence times from a common ancestor with H. sapiens

| **Species** | **Informative Classification** | **Estimated Divergence Times** | **Ascension #** |
| --- | --- | --- | --- |
| *Monosiga brevicolis* | Protist, Choanoflagellate | 1400 mya | XP_001746593 |
| *Caenorhabditis elegans* | Invertebrate, Protostome, Nematoda | 950 mya | NP_503425 |
| *Drosophila melanogaster* | Invertebrate, Protostome, Arthropoda | 950 mya | NP_477034 |
| *Ciona intestinalis* (sea squirt) | Invertebrate, deuterostome, Tunicata | 750 mya | XP_002131354 |
| *Branchiostoma floridae* (lancelet) | Invertebrate, deuterostome, Cephalochordata | 650 mya | XP_002614024 |
| *Salmo salar* (Atlantic salmon) | Vertebrate, Actinopterygii | 450 mya | NP_001133173, ACN12515, ACM08559 |
| *Danio rerio* (zebrafish) | Vertebrate, Actinopterygii | 450 mya | NP_998804, NP_991263, NP_998806 |
| *Xenopus tropicalis* (Western clawed frog) | Vertebrate, Amphibia | 360 mya | NP_998878, NP_001011156 |
| *Gallus gallus* (chicken) | Vertebrate, Aves, Phasianidae | 310 mya | NP_990859, NP_001004406 |
| *Sus scrofa* (domestic pig) | Vertebrate, Mammalia, Suidae | 66 mya | NP_001004043, NP_001020386, NP_001004031 |
| *Mus musculus* (domestic house mouse) | Vertebrate, Mammalia, Muridae | 66 mya | NP_031713, NP_031714, NP_062745 |
| *Homo sapiens* | Vertebrate, Mammalia, Hominidae | 0 | NP_005498, NP_068733, NP_006861 |

Estimates of divergence times among species were extrapolated from the following references: DeVries et al., 2006; King et al., 2008.
